# The efficacy of vitamin D supplementation in the management of childhood asthma: a systematic review and meta-analysis

**DOI:** 10.3389/fnut.2026.1842895

**Published:** 2026-05-19

**Authors:** Runze Li, Kaiyi Liu, Ning Chen, Wei Xu, Qi Cheng

**Affiliations:** Department of Pediatrics, Shengjing Hospital of China Medical University, Shenyang, China

**Keywords:** asthma, children, FEV1, lung function, vitamin D

## Abstract

**Background:**

Childhood asthma is a major public health concern. Current evidence regarding vitamin D supplementation in childhood asthma remains inconsistent, particularly with respect to pulmonary function. This study comprehensively evaluates the role of vitamin D supplementation in childhood asthma, with a particular focus on lung function.

**Method:**

PubMed, EMBASE, Web of Science, and the Cochrane Library were searched through October 31, 2025 for RCTs. Data were extracted and analyzed using FEV1 (%) as the primary outcome measure, and FVC (%), FEV1/FVC, PEF, FeNO, cACT, and IgE as secondary outcome measures.

**Results:**

Twelve RCTs (*n* = 1239) were included. We did not find evidence that vitamin D supplementation improves lung function [FEV1 (%): SMD: 0.39, 95% CI: –0.13 to 0.92; FVC (%): SMD: 0.13, 95% CI: –0.73 to 0.98; FEV1/FVC: MD: 0.56, 95% CI: –7.24 to 8.35; PEF: MD: -3.69, 95% CI: –9.71 to 2.34] or cACT scores (MD: 0.16, 95% CI: –0.55 to 0.88), nor does it reduce FeNO or IgE levels (SMD: –0.14, 95% CI: –0.61 to 0.33) in children with asthma.

**Conclusion:**

Vitamin D supplementation showed no overall benefit for lung function, IgE levels, FeNO, or cACT scores in children with asthma.

**Systematic review registration:**

https://www.crd.york.ac.uk/prospero/, identifier CRD420251270660.

## 1 Introduction

Asthma is one of the most common chronic respiratory diseases in children, imposing a substantial burden on child health and the global economy (1). According to a 2021 report from the U.S. Centers for Disease Control and Prevention (CDC), the prevalence of asthma among children aged 0–4, 5–11, and 12–17 years was 1.9 7.5 and 8.7%, respectively. In 2021, the mortality rate from childhood asthma reached 2.0 per million population. Lung function is a key indicator of asthma severity, and its importance in both clinical and epidemiological studies has increasingly been recognized. Regular monitoring of lung function is considered beneficial for the management of pediatric asthma (2, 3).

Vitamin D is a fat-soluble steroid hormone. It is mainly synthesized in the skin after exposure to ultraviolet radiation and can also be obtained from dietary sources. Its classical and most widely recognized core function is the maintenance of bone health, mainly through the regulation of calcium and phosphorus metabolism (4). In recent years, the immunomodulatory effects of vitamin D have been increasingly appreciated (5–9). Most immune cells, including macrophages, dendritic cells (DCs), T cells, and B cells, express the vitamin D–activating enzyme (1α-hydroxylase, CYP27B1) and the vitamin D receptor (VDR). Activated vitamin D (1,25-dihydroxyvitamin D, 1,25D) can directly regulate the functions of these cells as a signaling molecule (5, 6). The efficacy of vitamin D supplementation in the prevention an (or) treatment of various diseases, including asthma, respiratory infections, autoimmune diseases, and cancer, has attracted considerable attention (5–7, 10, 11).

Observational studies have shown that vitamin D deficiency in children is associated with an increased risk of asthma, and that lower vitamin D levels in children with asthma are linked to disease exacerbation and progressive decline in lung function (12–15). Moreover, preclinical studies on asthma indicate that vitamin D can attenuate airway inflammation and reduce airway remodeling through mechanisms such as immunomodulation and direct effects on airway smooth muscle cells (16–18). Taken together, these findings strongly suggest that vitamin D supplementation may help improve asthma control in children, particularly by enhancing lung function.

To date, several randomized controlled trials (RCTs) have evaluated the efficacy of vitamin D supplementation in children with asthma. Recent meta-analyses have shown that vitamin D supplementation in this population helps reduce asthma exacerbations (19–21). However, evidence regarding lung function, primarily forced expiratory volume in 1 s (FEV1), remains inconsistent. Some RCTs have reported significant improvements in children with asthma following vitamin D supplementation (22–24), whereas meta-analyses indicate that vitamin D intervention has no significant effect on lung function and may even worsen it (19–21, 25, 26). Furthermore, few studies have reported significant improvements in lung function after vitamin D supplementation, which stands in contrast to findings from preclinical studies.

Our study aims to investigate the effect of vitamin D supplementation on asthma management in children. Given the conflicting evidence, we specifically focus on the impact of vitamin D supplementation on lung function in children with asthma.

## 2 Materials and methods

### 2.1 Protocol and registration

This meta-analysis was conducted in accordance with the Preferred Reporting Items for Systematic Reviews and Meta-Analyses (PRISMA) guidelines. The study protocol was prospectively registered with PROSPERO (registration number: CRD420251270660) on 24 December 2025, before study selection, data extraction, risk-of-bias assessment, or quantitative synthesis commenced. The database search was completed before registration, and no additional searches were performed after registration.

### 2.2 Search strategy

Two investigators independently searched four major databases (PubMed, Web of Science, Embase, and Cochrane Library). According to our preregistered search strategy, databases were searched for articles published up to 31 October 2025, with no restrictions on the start date of the search. The search terms included: “Vitamin D,” “asthma,” “children,” “random controlled trials” (Supplementary Table 1). There were no language restrictions on the search.

The screening process comprised two phases in EndNote 21 (Clarivate Analytics). First, two investigators (R-ZL and K-YL) independently evaluated eligibility by reviewing the title and abstract. They then assessed the full text of the remaining articles against the inclusion criteria. Disagreements at any stage were resolved through discussion and input of a third investigator (QC) when necessary. No restrictions were applied regarding study settings or comparator groups. During the literature retrieval process, we did not contact study authors. For gray literature, we only reviewed several conference abstracts related to the included studies as supplementary sources. These abstracts were not treated as independently eligible studies. Instead, limited information from them was used solely to cross-check or supplement findings reported in the corresponding full-text articles (27, 28), and the source of each such datum was explicitly identified in the relevant analyses or discussion.

### 2.3 Search criteria

Inclusion criteria: Children/adolescents (< 18 years old); studies comparing the effects of vitamin D with placebo; patients clinically diagnosed with asthma; randomized controlled trial design; and studies reporting complete data for at least one of the following outcomes: FEV1 (%), forced vital capacity (FVC) (%), FEV1/FVC, peak expiratory flow (PEF), fractional exhaled nitric oxide (FeNO), childhood asthma control test (cACT) score, or IgE;

Exclusion criteria: adult population; non-interventional studies such as cohort studies or case-control studies; reviews, meta-analyses, expert consensuses, or clinical trial protocols; publications without full text or with data that cannot be extracted.

### 2.4 Risk of bias

The quality assessment tool used was ROB 2.0, published by Cochrane, which evaluates the risk of bias in studies across five domains: bias arising from the randomization process, bias due to deviations from intended interventions, bias due to outcome measurement, bias due to missing outcome data, and bias due to selective reporting of outcomes. The included studies were rated as having low risk of bias, high risk of bias, or as raising some concerns. If the assessments for all five domains are low risk, the overall risk of bias is considered low (Low risk of bias). If any single domain is judged to be at high risk, or if multiple domains raise potential concerns, the overall risk of bias is considered high (High risk of bias). RCTs that do not fall into either of the above categories are judged as having some concerns regarding risk of bias (Some concerns).

### 2.5 Data extraction

Two investigators (R-ZL and K-YL) independently extracted data from eligible studies. Extracted data included first author, publication year, country, sample size, vitamin D dosage, duration, cumulative dose, co-intervention and outcomes. Primary outcome metrics was FEV1 (%), and secondary outcome metrics were FVC (%), FEV1/FVC, PEF, FeNO, CACT score and IgE. Any disagreements were resolved through consultation with the third independent reviewer (QC). Note: Authors of studies with incomplete outcome data were not contacted for raw data.

### 2.6 Statistical analysis

We conducted statistical analyses of the data using Review Manager (version 5.4).

For continuous outcomes reported as median (Q1–Q3), we estimated the corresponding mean and SD using the published methods of Luo et al. (29) and Wan et al. (30), implemented in the web-based calculator developed by Tong and colleagues.^1^ This procedure was applied only to Jat et al. (31) and Thakur et al. (32). To minimize bias, we used this approach only when the reported quartiles suggested an approximately symmetric distribution; clearly skewed data were not converted and were summarized narratively.

For continuous variables, we applied fixed/random effects model to calculate the mean difference (MD), standardized mean difference (SMD), and corresponding 95% confidence intervals (CIs). The chi-square and I^2^ tests were used to measure study heterogeneity. If *P* heterogeneity > 0.05, and I^2^ > 50%, a random-effects model was used.

A *P* < 0.05 was considered statistically significant. Heterogeneity across studies was assessed using the I^2^ statistic. In order to explore the impact and heterogeneity of primary outcome, we conducted subgroup analyses of FEV1 (%) based on the duration of vitamin D supplementation, baseline vitamin D levels, use of corticosteroids, and daily dose equivalents. Notably, subgroup analyses were not prespecified in a prospectively published protocol. Given the considerable heterogeneity in supplementation regimens across the included studies and the absence of universally accepted thresholds for dose- or duration-based categorization in this context, subgroup boundaries were defined according to the observed distributions of dose and duration among the included studies. This approach was chosen to minimize arbitrariness in cut-point selection and to ensure an adequate number of studies within each subgroup. These subgroup analyses should therefore be regarded as exploratory. In addition, we conducted sensitivity analyses and assessed publication bias for the primary outcome indicators using StataSE 15 (64-bit).

Continuous outcomes were pooled using MD when studies reported results on the same scale and SMD when outcomes were reported using different units or scales. According to the Cochrane Handbook for Systematic Reviews of Interventions (section 10.5.2), post-intervention values and change-from-baseline values were not combined in the same meta-analysis when SMD was used. Therefore, for outcomes synthesized using SMD, separate meta-analyses were conducted for post-intervention values and change-from-baseline values. Notably, to avoid introducing additional assumptions, we did not derive the SD of change-from-baseline values from baseline and post-intervention data using assumed correlation coefficients (Cochrane Handbook, section 6.5.2.8). Accordingly, change-from-baseline analyses were restricted to studies that directly reported change scores. Furthermore, some studies included in our analysis reported both change-from-baseline and post-intervention values for certain outcome measures. When outcome measures were analyzed using MD as the effect measure, the two types of values from the same study were analyzed separately. Supplementary Figure 1 presents a detailed flowchart of the analytical process for post-intervention values and change-from-baseline values.

## 3 Results

### 3.1 Study characteristics

**FIGURE 1:**
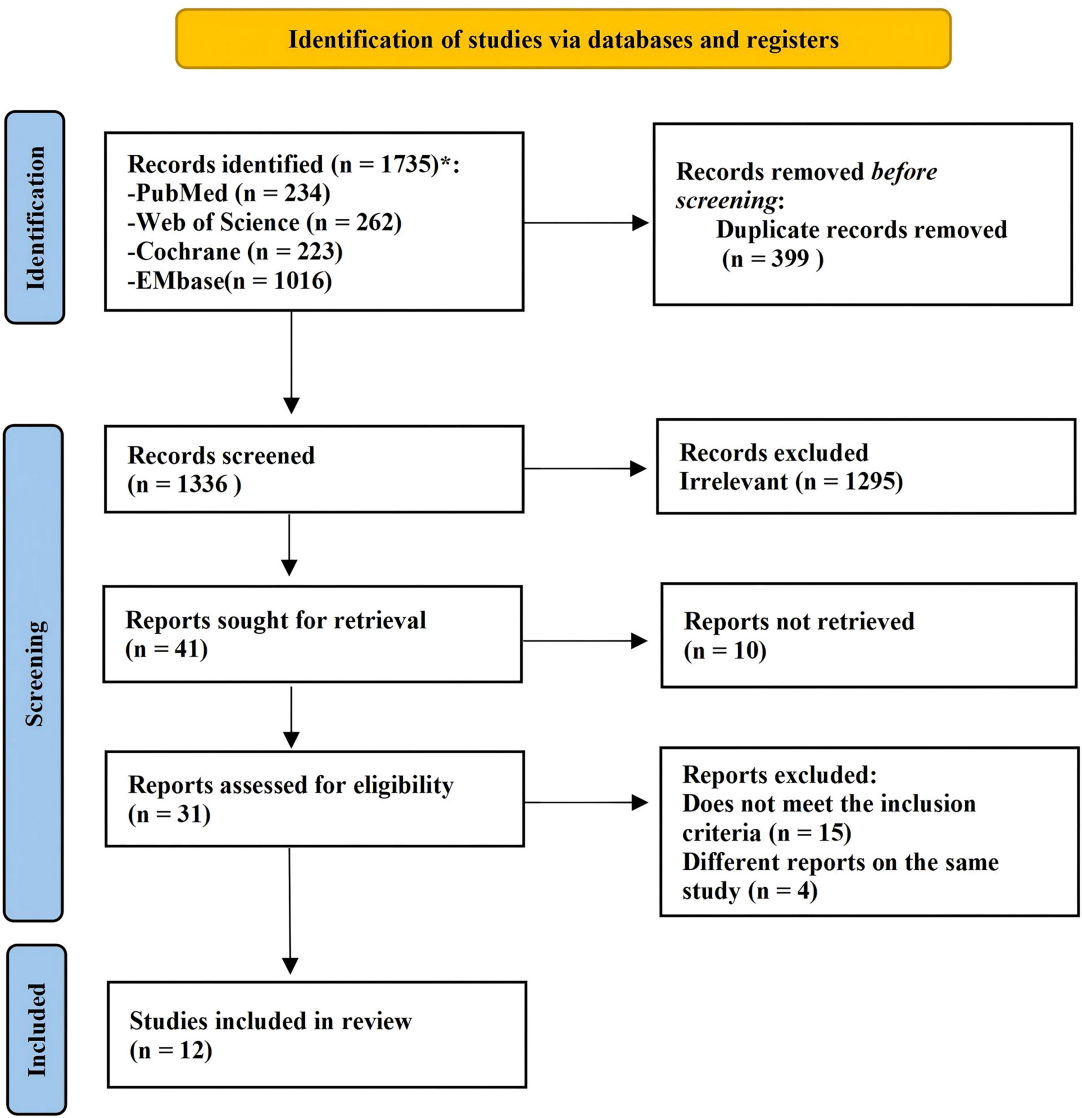
PRISMA flow chart was adapted from the PRISMA 2020 flow diagram template downloaded from https://www.prisma-statement.org/prisma-2020- flow-diagram. The template is licensed under CC BY 4.0 (https://creativecommons.org/licenses/by/4.0/).

According to our search strategy, a total of 1,735 studies were retrieved from four databases (Supplementary Table 1). After excluding 399 duplicates, 1,295 studies were initially removed due to age range mismatch, non-RCT design, classification as reviews or conference abstracts, other non-eligible literature types, or clearly irrelevant topics. Subsequently, 29 studies were excluded after full-text screening, resulting in 12 studies being included in this meta-analysis (Figure 1 and Supplementary Table 2). The characteristics of the included studies are summarized in Tables 1, 2. All included studies were RCTs and enrolled children with asthma aged 4–18 years. A total of 1,239 children with asthma were included, of whom 627 were assigned to the vitamin D supplementation group and 612 to the placebo group. More than half of the included studies (7/12) (22–24, 31–34) were conducted in Asia: India (2/12) (31, 32), China (2/12) (23, 24), Japan (33), Pakistan (22), and Israel (34). In addition, studies were conducted in Poland (2/12) (35, 36), the United States (37), Ireland (38), and Türkiye (39). The dosage of vitamin D supplementation ranged from 500 to 4,000 IU/day, with most studies using doses below 1,000 IU/day (6/12) (23, 24, 33, 35, 36, 39), while others used 2,000 IU/day (3/12) (32, 34, 38), 1,000 IU/day (2/12) (22, 31), and 4,000 IU/day (1/12) (37). The duration of vitamin D supplementation ranged from 9 days to 12 months, with half of the studies administering vitamin D for no more than 3 months (6/12) (22–24, 32–34). 9 studies reported baseline vitamin D levels in children with asthma (31–39), and in most of these studies, patients had vitamin D insufficiency (4/9) (33, 34, 37, 38) or even deficiency (3/9) (31, 32, 39). The primary outcome measures we extracted were FEV1 (L) and FEV1%, and 11 of the 12 studies reported FEV1 (22–24, 31–33, 35–39). In addition, the secondary outcome measures reported in the 12 articles were relatively heterogeneous and included FVC (3/12) (22, 31, 38), FEV1/FVC% (3/12) (24, 31, 38), PEF (3/12) (31, 33, 39), FeNO (2/12) (32, 34), cACT score (5/12) (22, 31–33, 38), and IgE level (2/12) (34, 39).

**TABLE 1:** Details of included studies.

Study (author, year)	Country	Sample size (VD/Control)	Age (years)	Dose (VD/Control)	Duration	Cumulative dose (VD/Control)	Co-intervention	Outcomes
Majak P, 2011 (35)	Poland	24/24	5–18	500 IU per day/vitamin D placebo	6-month	90,000 IU/NA	Budesonide 800 mg/d	FEV1
Forno E, 2020 (37)	United States	96/96	6–16	4000 IU per day/placebo capsules	48-week	1,344,000 IU/NA	Inhaled fluticasone propionate (88 μg twice per day in children aged 6–11 years and 110 μg twice per day in children ≥ 12 years)	Time to a severe asthma exacerbation; Cumulative fluticasone dose
Bar Yoseph R, 2015 (34)	Israel	19/19	6–18	14,000 IU per week/placebo	6-week	84,000 IU/NA	NA	PC20-FEV1; FeNO; IgE
Thakur C,2021 (32)	India	30/30	6–11	2000 IU per day/placebo	3-month	168,000 IU/NA	Budesonide 400 μg and formoterol 24 μg daily	cACT score; FEV1; FeNO
Kerley CP, 2016 (38)	Ireland	17/22	6–16	2000 IU per day/placebo	15-week	210,000 IU/NA	NA	cACT score; FEV1; FVC; FEV1/FVC
Tachimoto H,2016 (33)	Japan	54/35	6–15	800 IU per day/placebo	2-month	48,000 IU/NA	NA	C-ACT score; PEF;
Bashir T, 2023 (22)	Pakistan	87/88	6–14	1000 IU per day/placebo	12-week	84,000 IU/NA	NA	FEV1; FVC; cACT score
Majak P, 2009 (36)	Poland	18/18	6–12	1000IU per week/NA	3-month; 12-month	12,000 IU;48,000 IU/NA	Prednisone (Encorton 20 mg, Polfa Pabianice)	FEV1
Liu WJ, 2018 (24)	China	48/48	NA	800 IU per day/NA	9-day	7,200 IU/NA	Glucocorticoids and airway spasmolysis drugs	FEV1; FEV1/FVC
Zhu H, 2022 (23)	China	92/92	4–16	400 IU per day/NA	12-week	33,600 IU/NA	Salmeterol/Fluticasone	FEV1; PEF daily variation
Jat KR, 2021 (31)	India	125/125	4–12	1000 IU per day/placebo	9-month	270,000 IU/NA	Asthma treatment	FEV1; FVC; FEV1/FVC; PEF; cACT score
Baris S, 2014 (39)	Turkey	17/15	5–15	650 IU per day/NA	12-month	234,000 IU/NA	Subcutaneous immunotherapy (SCIT)	FEV1; PEF; lgE

**TABLE 2:** Baseline characteristics of included studies.

Study (author, year)		Age (years) Mean (SD)		FEV1 Mean (SD)		25(OH) D Mean (SD)		FVC Mean (SD)	
		I	C	I	C	I	C	I	C
Majak P, 2011 (35)		10.8 (3.2)	11.1 (3.3)	94.4% (13)	98.7% (12)	36.1 ng/mL (13.9)	35.1 ng/mL (16.9)	NA	NA
Forno E, 2020 (37)		9.9 (2.5)	9.7 (2.5)	93.9%(15.8)	90.6% (17.3)	22.5 ng/mL (4.6)	22.8 ng/mL (4.6)	NA	NA
Bar Yoseph R, 2015 (34)		13.5 (3.6)^1^	12.4 (3.6)	NA	NA	20.8 ng/mL (6.5)	20 ng/mL (7.1)	NA	NA
Thakur C,2021 (32)		9.0 (1.7)	8.7 (1.6)	75.3% (26.5)	75.6% (15.7)	15.8 ng/mL (8.2)	16.5 ng/mL (9.9)	NA	NA
Kerley CP, 2016 (38)		10 (6–12)^2^	7 (7–10)^2^	105% (92–114)^2^	96% (90–104)^2^	58 nmol/l (39–69)^2^	51 nmol (39–64)^2^	94.5% (87–101)^2^	93% (85–98)^2^
Tachimoto H,2016 (33)		10.0 (2.4)	9.8 (2.2)	88% (83–91)^2^	86% (84–91)^2^	28.5 ng/mL (23–33)^2^	29ng/mL (25–35)^2^	97% (91–106)^2^	97% (89–104)^2^
Bashir T, 2023 (22)		10.2 (2.1)	10.0 (2.3)	2.00 L (0.25)	2.01 L (0.22)	NA	NA	2.10 L (0.23)	2.08 L (0.21)
Majak P, 2009 (36)		NA	NA	93% (92–95)^4^	95% (92–97)^4^	32.0 ng/mL (3.1)^5^	31.3 ng/mL (3.4)^5^	NA	NA
Liu WJ, 2018 (24)		5.9 (3)	6.1 (2.8)	1. 28 L (0.33)	1. 16 L (0.26)	NA	NA	NA	NA
Zhu H, 2022 (23)		6.9 (3.2)	6.4 (2.1)	1.18 L (0.26)	1.22 L (0.30)	NA	NA	NA	NA
Jat KR, 2021 (31)		8.2 (2.3)	7.8 (2.2)	92.5% (21.7)^3^	97.0% (17.5)^3^	11.6 ng/mL (4.6)	10.8 ng/mL (4.4)	92.7% (21.7)^3^	94.6% (17.0)^3^
Baris S,2014 (39)		9.2 (2.6)	8.8 (1.1)	92% (14)	89% (14)	19 ng/mL (9.0)	20 ng/mL (12)	NA	NA
**FEV1/FEC Mean (SD)**		**PEF Mean (SD)**		**FeNO (ppb) Mean (SD)**		**IgE (IU/mL) Mean (SD)**		**cACT score Mean (SD)**	
**I**	**C**	**I**	**C**	**I**	**C**	**I**	**C**	**I**	**C**
NA	NA	NA	NA	NA	NA	NA	NA	NA	NA
91.5% (9.3)	89.6% (10.1)	NA	NA	NA	NA	NA	NA	NA	NA
NA	NA	NA	NA	36.6 (39.1)	58.6 (54.7)	432.8 (465.7)	433.8 (455.0)	NA	NA
NA	NA	NA	NA	17 (10.8–31.5)^2^	16(9.8–41)^2^	NA	NA	18 (2.9)	15.5 (2.7)
96% (88–99)^2^	94% (89–97)^2^	NA	NA	NA	NA	138 (28–440)^2^	399 (106–679)^2^	19 (17–21)^2^	17 (14.3–19)^2^
88% (84–91)^2^	86% (82–91)^2^	94% (76–106)^2^	84% (73–93)^2^	NA	NA	601 (320–1121)^2^	306 (46–618)^2^	25 (23–27)^2^	26 (25–27)^2^
NA	NA	NA	NA	NA	NA	NA	NA	16.8 (2.0)^3^	17.1 (1.9)^3^
NA	NA	NA	NA	NA	NA	NA	NA	NA	NA
47. 76% (3.30)	52. 02% (2.70)	NA	NA	NA	NA	NA	NA	NA	NA
NA	NA	NA	NA	NA	NA	NA	NA	NA	NA
98.5% (10.9)^3^	99.3% (10.1)^3^	84.9% (27.8)^3^	86.3% (23.9)^3^	NA	NA	NA	NA	21.7 (4.2)	21.9 (3.6)
NA	NA	83% (17)	82% (8)	NA	NA	478 (545)	381 (275)	NA	NA

^1^*n* = 20;

^2^Median (first-third quartile);

^3^n unknown;

^4^95%CI;

^5^*n* = 17.

### 3.2 Primary outcome

#### 3.2.1 Effect of vitamin D supplementation on FEV1

Among the 12 studies included, 11 reported FEV1 (22–24, 31–33, 35–39). However, 2 of the studies only reported that supplementing vitamin D had no significant effect on FEV1 in children with asthma, but did not provide specific values (28, 33). Han et al. (28) is a conference abstract reporting a secondary analysis of patient data originally presented in Forno E, 2020. Therefore, only 9 studies were included in the analysis, encompassing 838 participants (420 receiving vitamin D and 418 receiving placebo). 6 studies included FEV1(% pred) (31, 32, 35, 36, 38, 39), and 3 studies included FEV1 (L) (22–24). Therefore, SMD was used as the effect measure for this outcome. Besides, 8 studies provided post-intervention FEV1 values for both patient groups (22–24, 31, 32, 35, 36, 39), while 3 studies reported changes from baseline in FEV1 (24, 31, 38). It should be noted that Kerley et al. (38) reported changes from baseline of FEV1 in their manuscript as median (Q1–Q3). We subsequently obtained data presented as Mean and SD from a conference abstract published in 2014 and used these data for further analysis (27). In the analysis of other secondary outcomes (cACT, FVC and FEV1/FVC), the data source in the Kerley CP, 2016 study is consistent with this.

**FIGURE 2:**
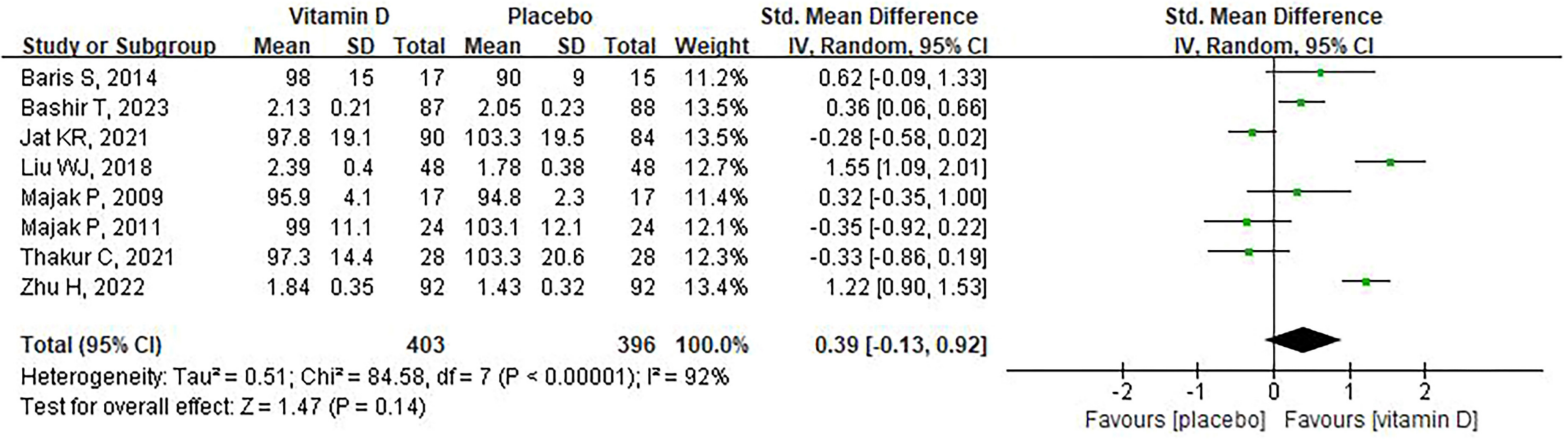
The impact of vitamin D supplementation on FEV1 (post-intervention values).

According to our statistical analysis strategy, the summary of post-intervention data showed that there was no significant difference between the placebo group and vitamin D group [SMD: 0.39, 95% CI: –0.13 to 0.92, heterogeneity:(*P* < 0.0001, *I*^2^ = 92%), Figure 2]. The analysis of the change-from-baseline data produced consistent results [SMD: 0.21, 95% CI: –0.64 to 1.06, heterogeneity: (*P* < 0.0001, *I*^2^ = 91%), Supplementary Figure 2].

**FIGURE 3:**
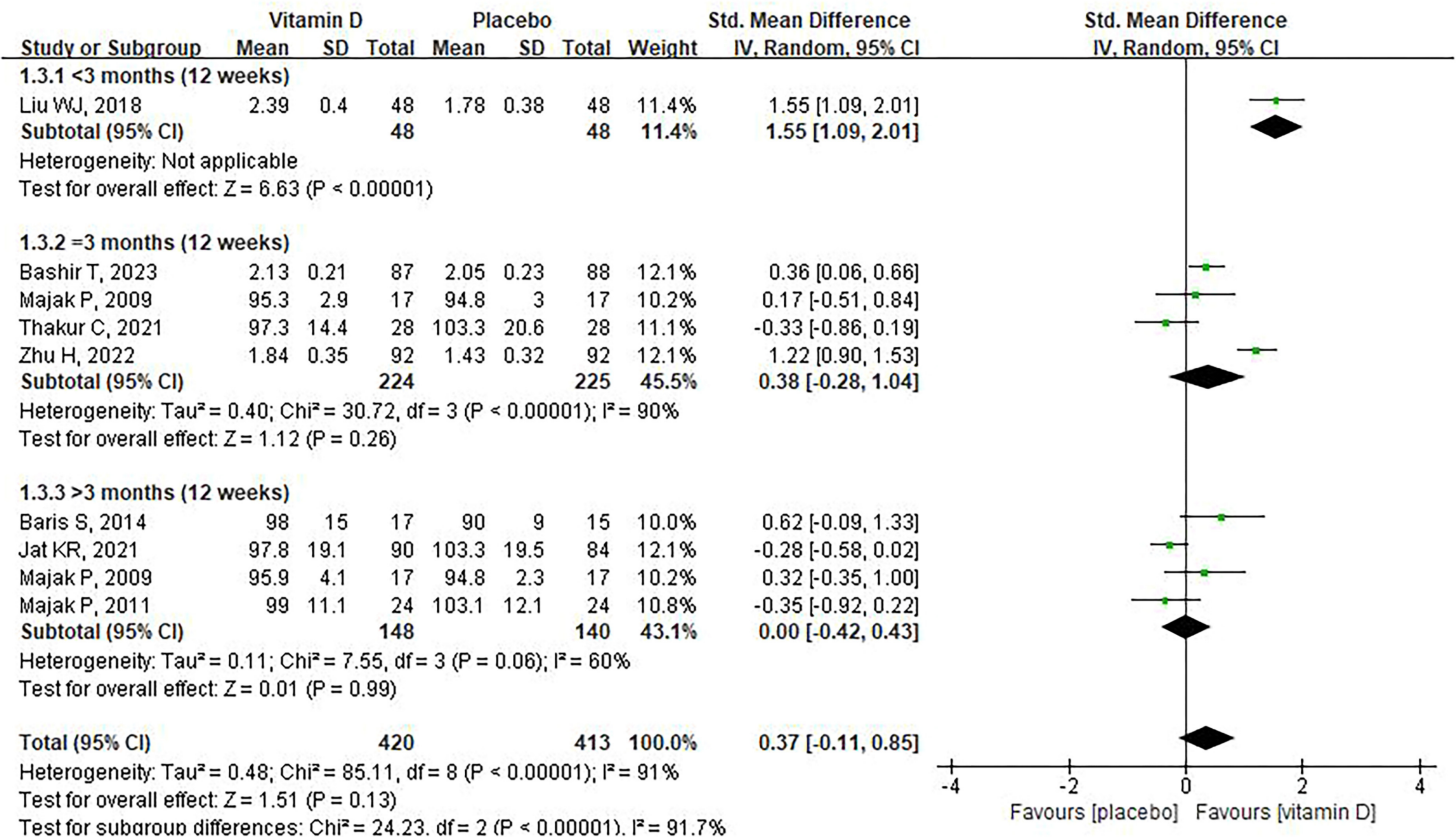
The impact of vitamin D supplementation on FEV1 based on duration of supplementation.

**FIGURE 4:**
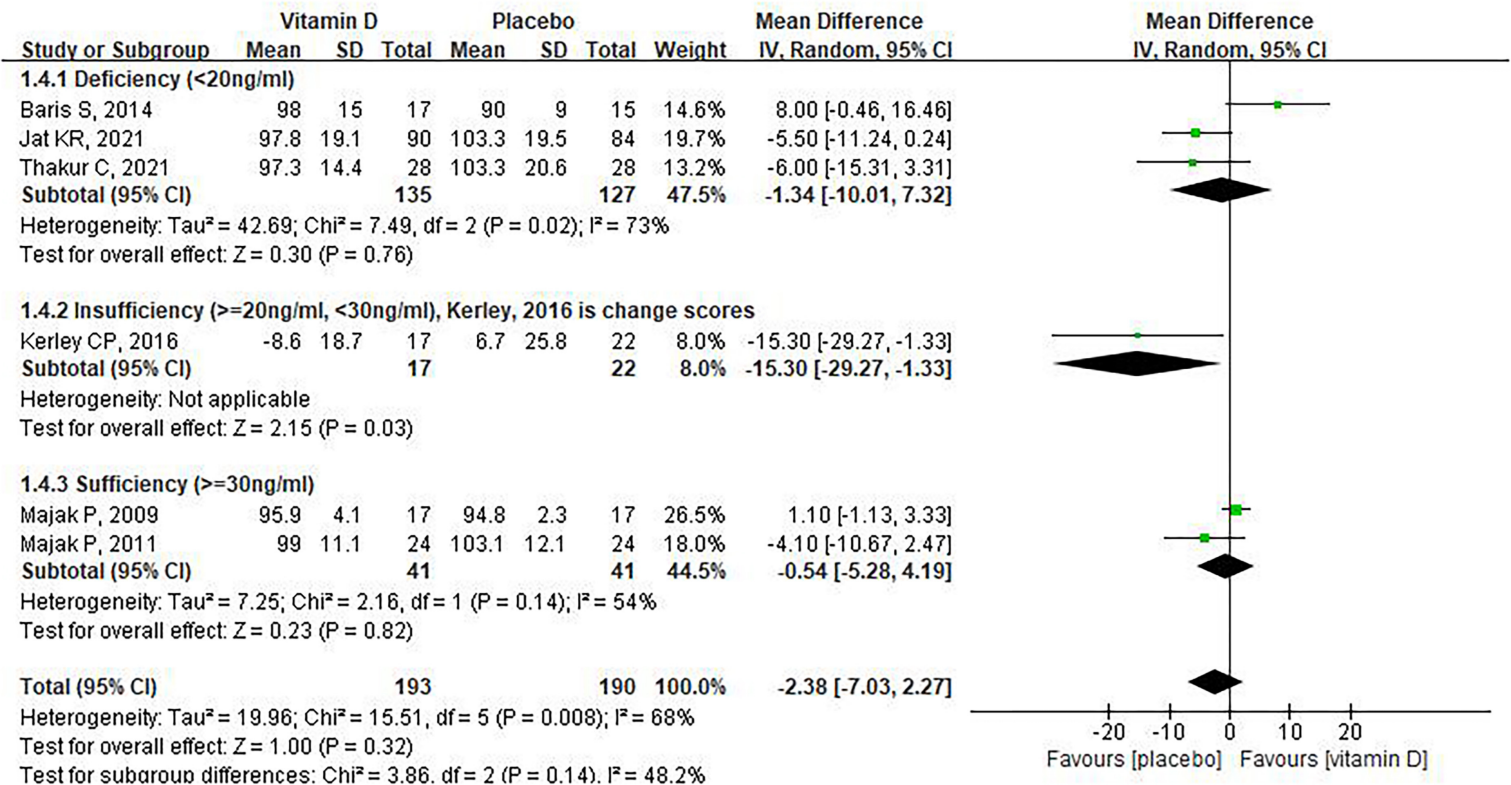
The impact of vitamin D supplementation on FEV1 based on the baseline of Vitamin D level.

**FIGURE 5:**
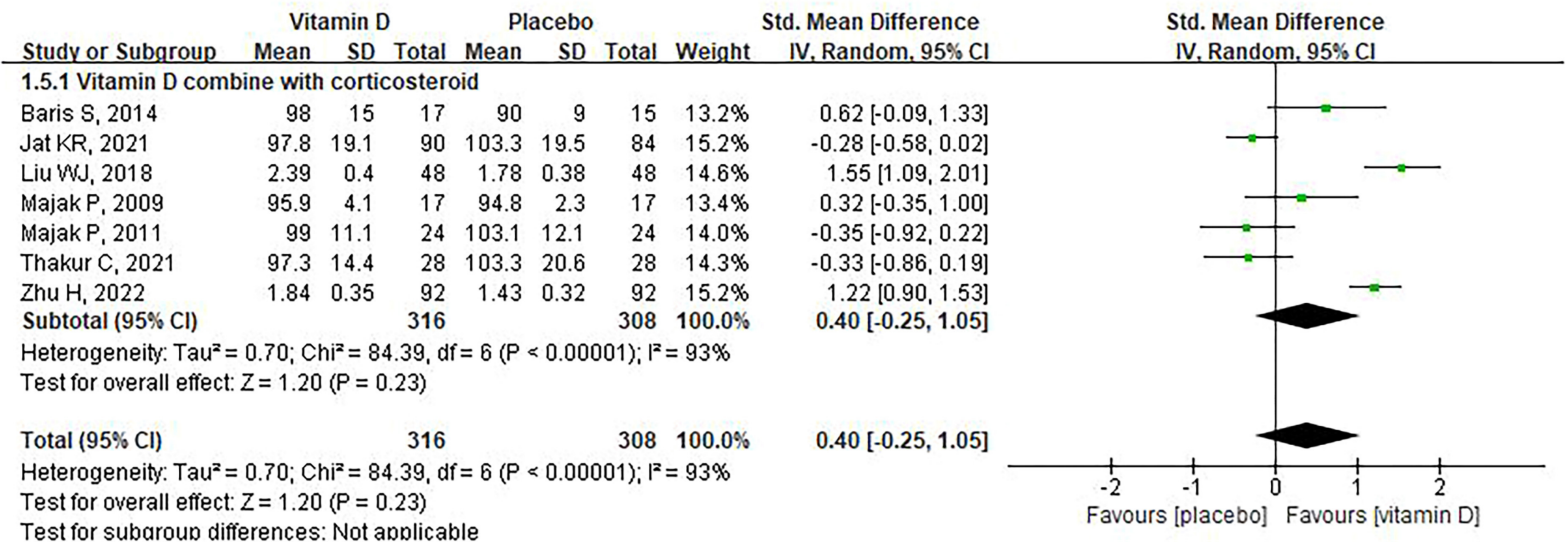
The impact of vitamin D supplementation on FEV1 based on the use of corticosteroid.

**FIGURE 6:**
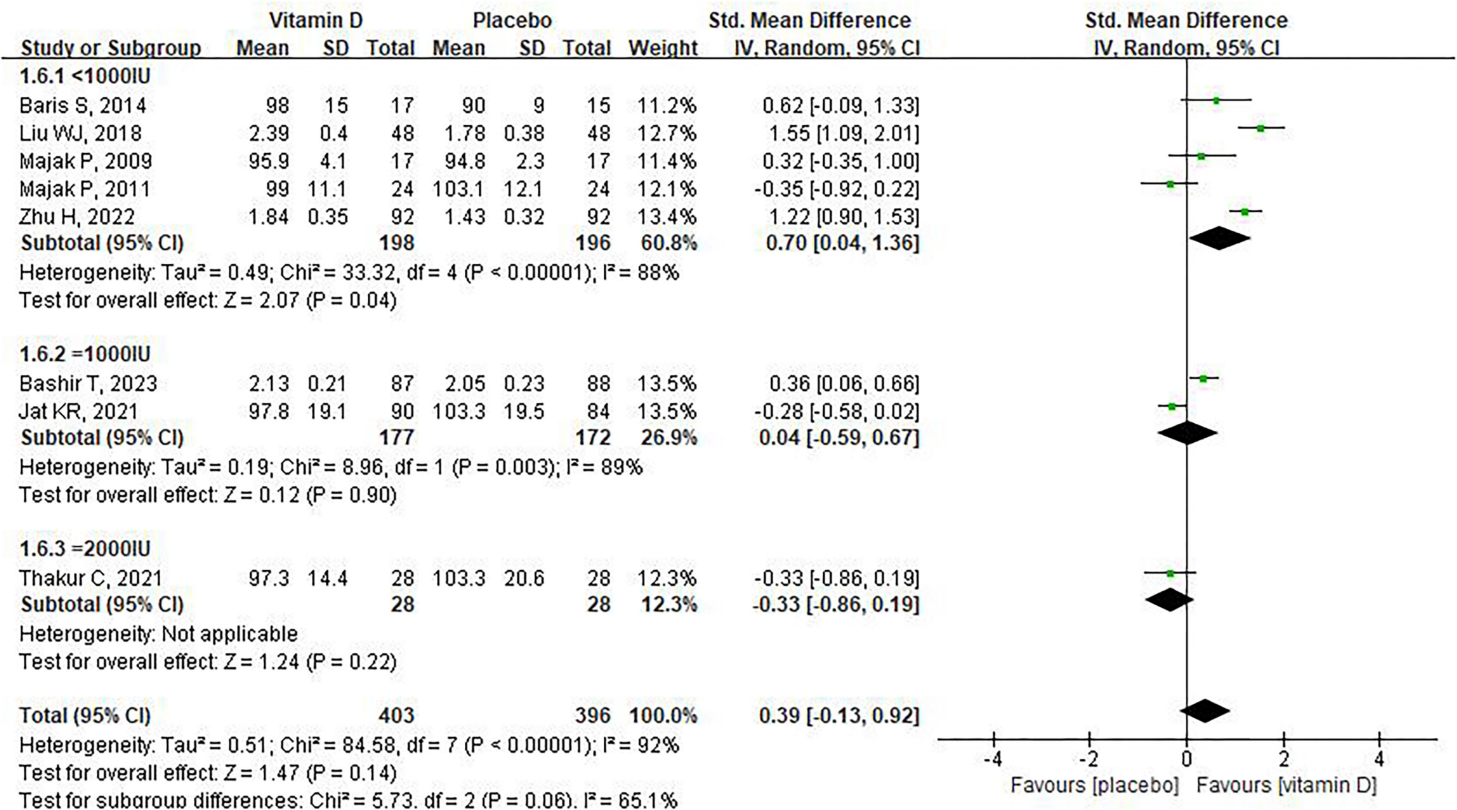
The impact of vitamin D supplementation on FEV1 based on the daily dose equivalent.

We further conducted the subgroup analysis on FEV1. For supplement duration of vitamin D, Liu et al. observed a significant increase in FEV1 after supplementing severe asthma children with vitamin D for 9 days (SMD:1.55 95% CI: 1.09–2.01, Figure 3), whereas no difference in FEV1 was observed between the placebo and vitamin D groups when the supplementation time was 3 months or more [ = 3 months: SMD: 0.38, 95% CI: –0.28 to 1.04, heterogeneity: (*P* < 0.00001, *I*^2^ = 90%); > 3 months: SMD: 0.00, 95% CI: –0.42 to 0.43, heterogeneity: (*P* = 0.06, *I*^2^ = 60%), Figure 3]. For the baseline of vitamin D levels, six of the nine studies reported baseline vitamin D levels in children, which we classified as deficiency (< 20 ng/mL), insufficiency (≥ 20 ng/mL, < 30 ng/mL) and sufficiency (> 30 ng/mL). Subgroup analysis indicated that, compared with placebo, vitamin D supplementation had no significant effect on FEV1 in asthmatic children, regardless of whether they had deficient or sufficient vitamin D levels (Figure 4). Moreover, for asthmatic children with insufficiency vitamin D levels, vitamin D supplementation could reduce FEV1 compared to the control group (MD: -15.3, 95% CI: -29.27 to –1.33). However, it should be noted that this result was based on data from only one study. In addition, the combined use of vitamin D and corticosteroid does not have a significant effect on FEV1 in children with asthma (Figure 5). Finally, regarding the equivalent daily doses of vitamin D, a daily intake of less than 1000 IU was associated with improved FEV1 in children with asthma [SMD:0.70, 95% CI: 0.04–1.36, heterogeneity: (*P* < 0.00001, *I*^2^ = 88%), Figure 6], whereas higher daily doses of vitamin D did not exert a significant effect on FEV1 [1000IU: SMD: 0.04, 95% CI: –0.59 to 0.67, heterogeneity: (*P* = 0.003, I^2^ = 89%); 2000IU: SMD: –0.33, 95% CI: –0.86 to 0.19, Figure 6].

In summary, our subgroup analyses indicated a potential benefit of vitamin D supplementation at doses < 1,000 IU or for durations < 3 months in children with asthma. However, given factors such as limited sample sizes in these subgroups, these results should be considered strictly exploratory and interpreted with great caution (see Discussion).

### 3.3 Second outcome

#### 3.3.1 FVC (%)

**FIGURE 7:**
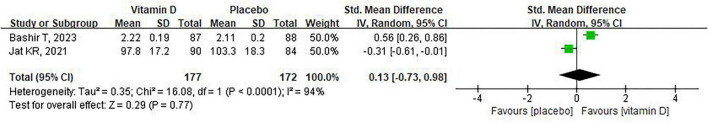
The impact of vitamin D supplementation on FVC (post-intervention values).

FVC is commonly used to assess airway resistance. Among the studies we included, three reported FVC (%) outcomes (22, 31, 38). Bashir et al. reported post-intervention values (22), Kerley et al. reported change-from-baseline (38), and Jat et al. reported both (31). Our analysis showed that, compared with the placebo group, vitamin D supplementation did not improve FVC (%) in children with asthma, whether based on change-from-baseline [SMD: –0.14, 95% CI: –0.42 to 0.14, heterogeneity: (*p* = 0.22, *I*^2^ = 35%), Supplementary Figure 3] or post-intervention values [SMD: 0.13, 95% CI: –0.73 to 0.98, heterogeneity: (*P* < 0.0001, *I*^2^ = 94%), Figure 7]. Notably, among these three studies, Bashir et al. reported that vitamin D supplementation significantly improved FVC in children with asthma, whereas the other two studies did not observe any difference compared with the control group.

#### 3.3.2 FEV1/FVC%

**FIGURE 8:**
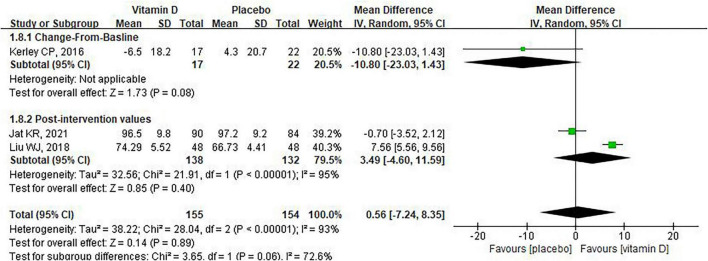
The impact of vitamin D supplementation on FEV1/FVC% (Jat KR, 2021 and Liu WJ, 2018 are post-intervention values).

Three studies reported the effect of vitamin D supplementation on FEV1/FVC% (24, 31, 38). Among them, Jat et al. and Liu et al. reported both change-from-baseline and post-intervention values (24, 31), whereas Kerley et al. reported only change-from-baseline values (38). To ensure the robustness of the findings, we conducted two separate analyses, respectively incorporating the change-from-baseline and post-intervention values from the studies by Jat et al. and Liu et al. The results of both analyses were consistent, indicating no significant effect of vitamin D supplementation on FEV1/FVC% in children with asthma [MD: 2.16, 95% CI: –7.72 to 12.04, heterogeneity: (*P* < 0.00001, *I*^2^ = 96%), Supplementary Figure 4; MD: 0.56, 95% CI: –7.24 to 8.35, heterogeneity: (*P* < 0.00001, *I*^2^ = 93%), Figure 8]. Notably, among these three studies, Liu et al. reported that vitamin D supplementation significantly improved FEV1/FVC% in children with asthma, whereas the other two studies found no difference compared with the control group.

#### 3.3.3 PEF

**FIGURE 9:**
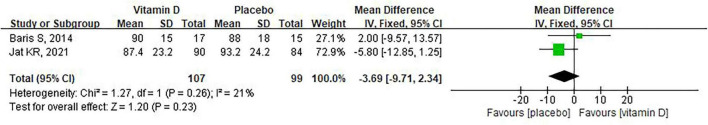
The impact of vitamin D supplementation on PEF (post-intervention values).

PEF is used to assess airway patency, and its continuous measurement can be used to monitor variable airway obstruction in children with asthma (2, 40). Two studies reported PEF (31, 39). Using the aforementioned methods, we conducted two analyses based on the change-from-baseline and post-intervention values reported by Jat et al., respectively, but neither analysis found a significant difference between the vitamin D group and the placebo group [MD: –2.69, 95% CI: –8.05 to 2.67, heterogeneity: (*P* = 0.37, *I*^2^ = 0), Supplementary Figure 5; MD: -3.69, 95% CI: -9.71 to 2.34, heterogeneity: (*P* = 0.26, *I*^2^ = 21%), Figure 9]. It is worth noting that the study by Tachimoto et al. (33) reported that after 2 months of 800 IU/day vitamin D supplementation, the proportion of patients with PEF < 80% at 6 months was significantly lower than that in the placebo group. Furthermore, Zhu et al. reported that daily oral administration of 400 IU of vitamin D for 12 consecutive weeks was significantly more effective than placebo in reducing the daily variability of PEF in children with asthma (23).

#### 3.3.4 FeNO

FeNO can non-invasively reflect the level of non-bacterial allergic inflammation and is associated with asthma severity (2, 41). Two studies assessed FeNO levels; however, neither observed significant changes in FeNO following vitamin D supplementation compared with placebo (32, 34). However, in the study by Thakur et al. (32), the researchers reported the post-intervention value of FeNO for the placebo group as [10 (8.3–21.8), median (Q1–Q3), *n* = 28], which does not conform to a normal distribution. Therefore, considering that the analysis of these converted data may introduce errors into the results, we did not perform a pooled analysis.

#### 3.3.5 cACT score

**FIGURE 10:**
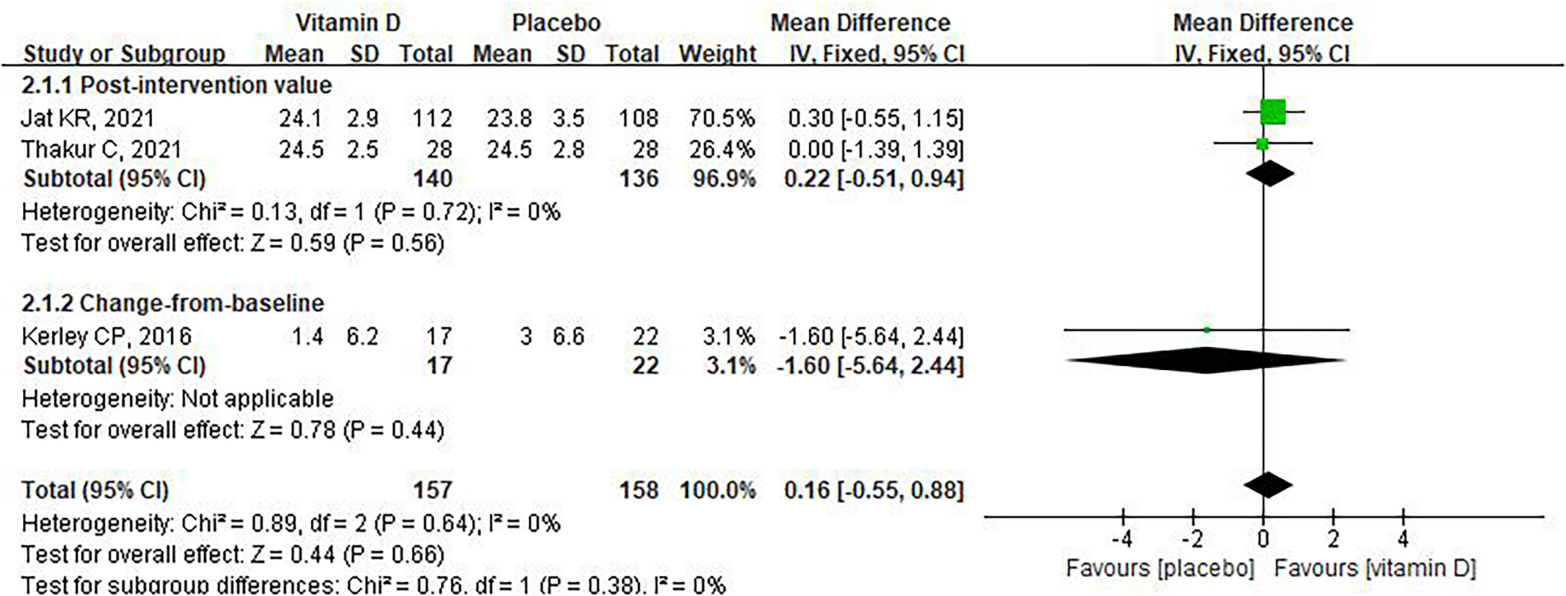
The impact of vitamin D supplementation on cACT score (Jat KR, 2021 is post-intervention values).

cACT is a brief questionnaire jointly completed by children and their parents that can rapidly and objectively assess asthma control in children aged 4–11 years with asthma (42). Among the studies we included, three reported cACT scores (31, 32, 38). Both of our analyses indicated that vitamin D supplementation did not significantly affect cACT scores [MD: 0.16, 95% CI: –0.55 to 0.88, heterogeneity: (*P* = 0.64, *I*^2^ = 0), Figure 10; MD: 0.29, 95% CI: –0.45 to 1.03, heterogeneity: (*P* = 0.54, *I*^2^ = 0), Supplementary Figure 6].

#### 3.3.6 IgE level

**FIGURE 11:**
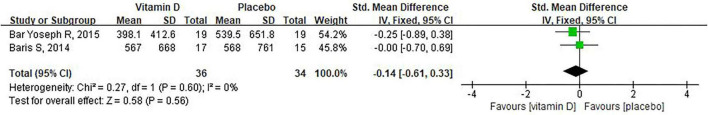
The impact of vitamin D supplementation on IgE level.

As an allergic disease, asthma is often accompanied by elevated IgE levels, and IgE plays an important role in its pathogenesis (43). Two studies reported IgE levels (34, 39), and our analysis did not identify a significant effect of vitamin D supplementation on IgE levels in children with asthma [SMD: –0.14, 95% CI: –0.61 to 0.33, heterogeneity: (*P* = 0.60, *I*^2^ = 0), Figure 11]. Additionally, in the study by Tachimoto et al., no change in IgE levels was observed after vitamin D supplementation in children with asthma, although the researchers did not report specific values (33).

#### 3.3.7 Risk of bias

**FIGURE 12:**
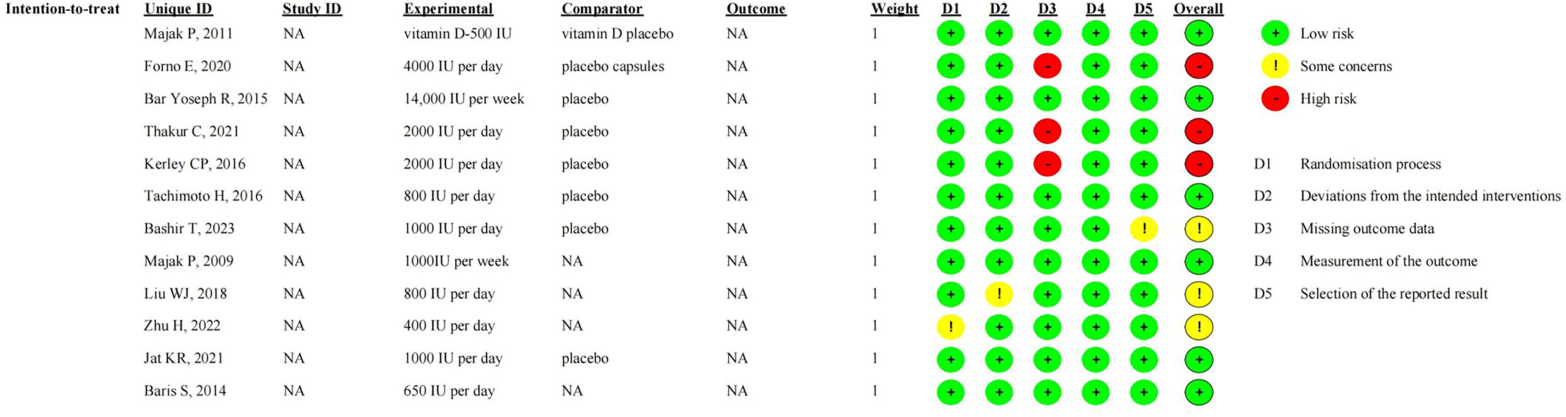
Risk of bias summary based on Cochrane Systematic Review Guidelines for each included study included in this review.

Risk of bias was assessed using ROB 2.0, as released by Cochrane. ROB 2.0 evaluates risk of bias across five domains: bias arising from the randomization process, bias due to deviations from intended interventions, bias due to missing outcome data, bias in measurement of the outcome, and bias in selection of the reported result. Among the 12 included studies, 3 were judged to have a high overall risk of bias, 3 were judged to raise some concerns regarding overall risk of bias, and the remaining 6 were considered to have a low overall risk of bias (Figure 12). In the studies with a high risk of bias, the primary issue was missing outcome data; for continuous outcomes primarily based on FEV1 or FEV1%, missing data in these 3 studies resulted in less than 95% of participant data being available, indicating a potential risk of bias. In the study by Bashir et al., the Methods section did not clearly state whether the data underlying the reported results were analyzed according to a pre-specified analysis plan, leading to some concerns about the overall risk of bias (22). The study by Liu et al. also raised some concerns in the Methods section because specific information on random sequence generation was not provided, with the authors only stating that the study was “randomized” (24). In addition, the study by Zhu et al. (23) was methodologically limited by insufficient reporting regarding whether the allocation sequence concealment was performed prior to participant enrollment and assignment of interventions.

#### 3.3.8 Sensitivity analysis

**FIGURE 13:**
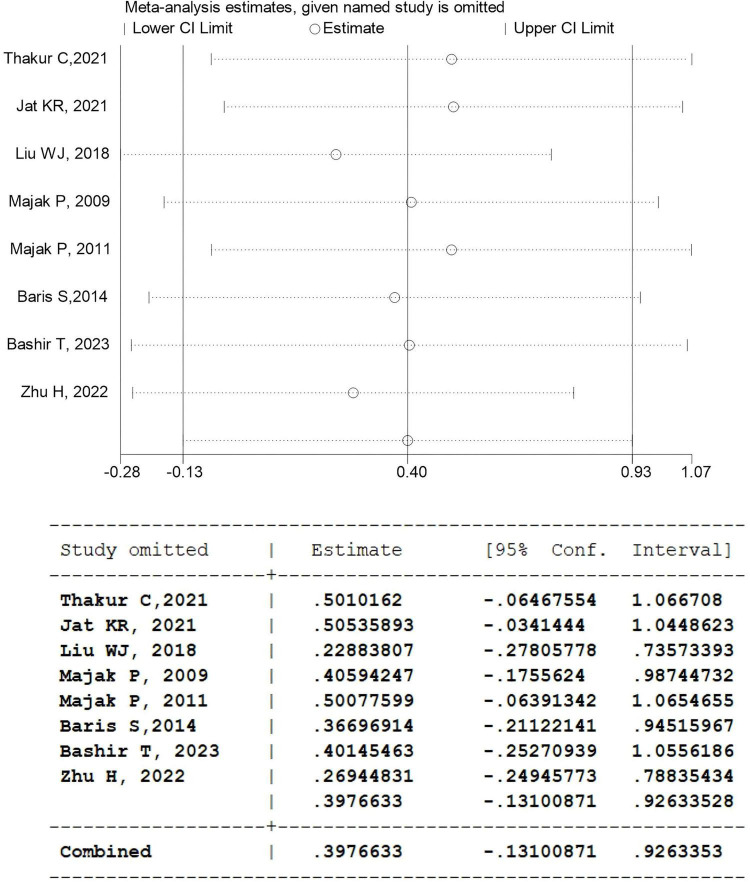
Sensitivity analysis for vitamin D supplementation on FEV1.

We used StataSE 15 (64-bit) to perform sensitivity analyses on the primary outcome indicators. The results indicated that, after sequentially excluding each individual study, the findings remained consistent with those from the pooled analysis of all studies, and no significant effect of vitamin D supplementation on FEV1 (%) in children with asthma was observed (Figure 13).

#### 3.3.9 Publication bias

**FIGURE 14:**
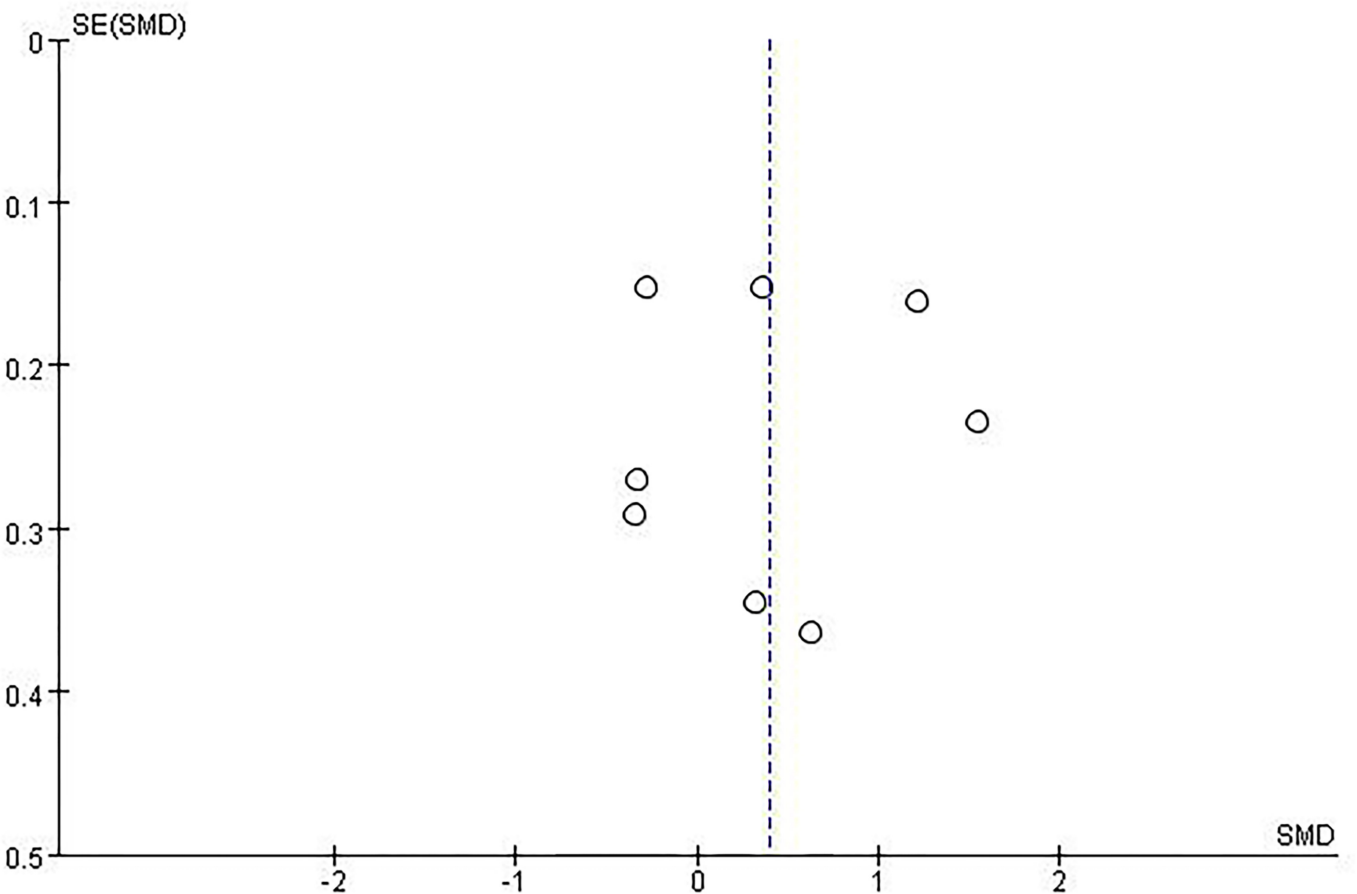
Funnel plot of primary outcome.

We used StataSE 15 (64-bit) to assess publication bias for the primary outcome (FEV1). Figure 14 shows the corresponding funnel plot. Visual inspection revealed some asymmetry, but Egger’s test did not indicate significant publication bias (*P* = 0.922; Supplementary Figure 7). Given that fewer than 10 studies were included, the results of both visual inspection and Egger’s test should be interpreted with caution. Accordingly, while there was no strong statistical evidence of publication bias, it cannot be definitively ruled out.

## 4 Discussion

In this meta-analysis, we did not observe a significant effect of vitamin D supplementation on lung function (FEV1, FVC, FEV1/FVC, PEF), inflammatory markers (IgE, FeNO), or asthma control status (cACT) in children with asthma. The above results are corroborated by numerous previous meta-analyses and systematic reviews. Specifically, previous analyses have reported that vitamin D supplementation in children with asthma does not significantly benefit on FEV1 (19, 25, 44), FVC (19), FEV1/FVC (26), FeNO (25, 26) or cACT score (25, 26). For IgE, a recent meta-analysis conducted by Abd et al. (45) showed that vitamin D supplementation had no significant effect on serum total IgE levels in patients with asthma (including both adults and children). Notably, although recent meta-analyses have suggested that vitamin D supplementation in children with asthma may decrease FEV1 and (or) FVC (20, 21, 26), our comprehensive synthesis of the available evidence does not support this conclusion.

The main clinical significance of this study is to help clarify the actual role of vitamin D in the management of pediatric asthma. Specifically, multiple commonly used indicators show no benefit, indicating that vitamin D should not be considered a routine adjunctive therapy for improving baseline lung function, inflammatory status, or asthma control in children with asthma. Meanwhile, these results should be interpreted in conjunction with previous meta-analyses suggesting that vitamin D supplementation may reduce asthma exacerbations in childhood (20, 21). Taken together, the existing evidence suggests that vitamin D does not appear to have significant benefits for stable asthma-related parameters, but it may still play a role in reducing acute exacerbations. This distinction is important for clinical decision-making, as it suggests that vitamin D supplements should not be expected to enhance routine asthma control in all children, but rather should be regarded as a supplementary strategy for specific patients, especially those with recurrent episodes.

Moreover, we specifically examined the impact of vitamin D supplementation on FEV1 (%) across different patient subgroups. It is important to emphasize that, although these subgroup analyses suggest a potential benefit of short-term or low-dose vitamin D supplementation on FEV1 in children with asthma, the small sample sizes and substantial heterogeneity limit the strength of the evidence. Moreover, these subgroup analyses were conducted *post hoc* and were not included in our PROSPERO registration. In addition, the dose- and duration-based subgroup categories were defined according to the empirical distribution of the included studies rather than being specified *a priori* in a protocol. Although this strategy was intended to reduce arbitrariness in cut-point selection and to minimize sparse-data issues, it cannot fully eliminate the risk of data-driven bias. Different cut-offs might have yielded different subgroup estimates, and multiple comparisons increase the likelihood of chance findings. Therefore, these results should be regarded as exploratory and hypothesis-generating rather than as definitive evidence of causality. In the following sections, we will interpret the results of the subgroup analyses with appropriate caution.

In fact, previous analyses have already examined the duration and dosage of vitamin D supplementation in patients with asthma. Regarding supplementation duration, two meta-analyses showed that supplementation for less than or equal to 6 months can significantly reduce the number of asthma exacerbations, whereas longer supplementation periods did not differ from the control group (20, 21). However, with respect to FEV1, the meta-analysis by Liu et al. (20) reported no difference in FEV1 between the supplementation and control groups, even in the subgroup with a vitamin D supplementation duration of less than or equal to 6 months. Our analysis found that short-term vitamin D supplementation (9 days) can significantly improve FEV1 in children with asthma, but because this subgroup included only one study, the conclusion requires further confirmation in future research. One possible explanation for this finding is that short-term vitamin D supplementation may rapidly correct vitamin D insufficiency or deficiency in most children, exert immunomodulatory effects, and directly act on airway smooth muscle (16–18), thereby appropriately attenuating airway inflammation and remodeling. As a result, asthma exacerbations may decrease, and improvements can be detected by FEV1 measurements within a short period. By contrast, once adequate vitamin D status is achieved, prolonged supplementation may not confer additional benefits. Over time, the beneficial effects of vitamin D on lung function may be offset by other competing factors affecting FEV1, such as disease fluctuations, medication adherence, and environmental exposures, so the improvement in FEV1 associated with vitamin D supplementation may gradually diminish to a non-significant level. Similar perspectives have been extensively discussed in the guidelines previously proposed by Heaney (46). Specifically, supplementation with nutrients such as vitamin D is most likely to confer benefits when individuals are in a deficient state, whereas the incremental benefits of further increasing intake once sufficiency has been achieved may be attenuated. Nevertheless, this finding does not negate the need for long-term vitamin D supplementation in children with asthma to maintain sufficient levels as observational studies have linked vitamin D insufficiency or deficiency to asthma exacerbations and reduced lung function (12–15).

In terms of supplementation dosage, a recent meta-analysis by Fedora et al. (21) reported that supplementation with a standard dose (47) of vitamin D can significantly reduce the frequency of acute exacerbations in children with asthma, whereas high doses of vitamin D showed no significant improvement in exacerbation frequency. Our analysis showed that only in the subgroup with a daily supplementation dose of < 1,000 IU did the vitamin D group exhibit a significant increase in FEV1 compared with the placebo group. However, no significant differences in FEV1 were observed between the vitamin D and placebo groups in the daily doses of 1,000 IU or 2,000 IU subgroups. These results suggest that the effect of vitamin D supplementation on improving FEV1 may also follow a threshold-effect pattern rather than a linear dose–response relationship. However, the underlying biological mechanisms behind the insignificant benefit of supplementing high-dose vitamin D on FEV1 remain unclear. One potential reason is that high vitamin D exposure may limit the sustained enhancement of its signal through steady-state feedback such as CYP24A1/FGF23 (48), which make its improvement on FEV1 relatively decline. Another hypothesis is that high doses of vitamin D may induce immune regulatory patterns distinct from those elicited by low doses, and that higher doses do not necessarily confer additional anti-inflammatory benefits. For example, experimental studies have shown that under certain conditions, active vitamin D can skew CD4^+^ T-cell responses toward the Th2 phenotype (5, 49), which may offset some of its anti-inflammatory effects and result in limited further improvement in FEV1. Moreover, high heterogeneity in the low-dose group and the relatively small number of studies in the high-dose groups leave the evidence uncertain, so the current findings are insufficient to demonstrate that low-dose vitamin D supplementation is truly superior to higher doses in improving lung function.

In addition, we did not identify any subgroup of children with vitamin D sufficiency, insufficiency or deficiency who showed significant improvement in FEV1 when stratified by baseline 25(OH) D levels. Notably, in the vitamin D–insufficient subgroup, FEV1 significantly decreased after vitamin D supplementation, although this subgroup included only one study. In the same study, the investigators also performed stratified analyses of children with asthma according to baseline vitamin D levels. They found that asthmatic children with vitamin D deficiency (< 50 nmol/L) experienced a significant decrease in FEV1/FVC after vitamin D supplementation, whereas those with higher vitamin D levels (> 50 nmol/L) benefited from supplementation, as evidenced by reductions in days of school missed and steroid requirements (38). Finally, we analyzed the effect of the combined use of vitamin D and corticosteroids on FEV1. Preclinical studies suggest that vitamin D can counteract steroid resistance and enhance corticosteroid efficacy (17). Proposed mechanisms include increasing glucocorticoid receptor (GR) binding and histone H4 acetylation at the glucocorticoid response element (GRE) of the MAPK phosphatase 1 (MKP-1) promoter in monocytes, promoting GR nuclear translocation in airway epithelial cells, facilitating IL-10 secretion by lymphocytes and inhibiting Th17 responses, and suppressing the pro-inflammatory phenotype of neutrophils (17, 50–52). Indeed, this combination has been shown to significantly reduce exacerbations in children with asthma (21, 53). However, our analysis did not identify a significant improvement in FEV1 with the concomitant use of vitamin D and corticosteroids, suggesting that the potential synergistic effects of vitamin D and corticosteroids may not be reflected in FEV1 outcomes in existing clinical studies.

Notably, a recent meta-analysis reported that vitamin D supplementation significantly improved FEV1% in patients with asthma whose baseline FEV1% was below 70% (20). However, among the studies included in our analysis, most participants had a baseline FEV1% greater than 80%, and only one study enrolled patients with baseline FEV1% below 80% but above 70%. In that study, no significant difference in FEV1 was observed between asthmatic children receiving standard asthma therapy alone and those receiving additional vitamin D supplementation (32). Taken together, the current analysis is insufficient to clearly determine whether the therapeutic effects of vitamin D supplementation differ among asthmatic children with different baseline FEV1 levels. Further well-designed studies are needed to address this issue.

In this analysis, we selected lung function the main indicator of concern for several reasons. First, lung function parameters are objective, reproducible, and central to the pathophysiology of asthma. Second, as noted in the introduction, findings from some existing RCTs and meta-analyses regarding lung function are inconsistent with those of observational and preclinical studies. Third, the existing meta-analysis on the impact of VD supplementation on lung function in children with asthma is not sufficiently comprehensive, and the evidence needs to be updated. Moreover, FEV1 is the most commonly reported lung function indicator in RCTs, so we designated FEV1 as the primary outcomes for this meta-analysis. Nevertheless, FEV1 is fundamentally a surrogate endpoint, and its correlation with outcomes of direct importance to patients and their families may be only modest. From a clinical perspective, the goals of asthma management in children extend considerably beyond improvements in FEV1 percent predicted. The recently published Core Outcome Measures sets for pediatric Severe Asthma (COMSA) (54) recommends that all pediatric asthma trials routinely measure not only FEV1 but also the annualized severe exacerbation rate, maintenance oral corticosteroid use, the Pediatric Asthma Quality of Life Questionnaire (PAQLQ), and ACT. Notably, except for asthma exacerbations and cACT scores, other indicators are rarely reported in RCTs, which limits the feasibility of pooled analysis. Furthermore, given that a recent systematic review has comprehensively examined asthma exacerbation frequency in the context of vitamin D supplementation (21), our meta-analysis focused on the effects of vitamin D supplementation on lung function in children with asthma, while also paying additional attention to asthma control-related indicators such as FeNO and cACT scores. When interpreting the findings of this meta-analysis, it is important to recognize that improvements in FEV1 may not reflect meaningful clinical outcomes in children with asthma.

Finally, in addition to the direct effects of supplemental vitamin D on children with asthma, prenatal maternal vitamin D supplementation is also thought to potentially reduce the risk of asthma or recurrent wheezing by modulating the fetal immune system and promoting lung maturation, thereby contributing to the management of childhood asthma (17). Recent evidence suggests that the sphingolipid pathway may play an important role in improving offspring respiratory health through maternal vitamin D supplementation (55). To date, several clinical studies have examined the impact of maternal vitamin D supplementation during pregnancy on the subsequent risk of wheezing in children, but their conclusions have been inconsistent (56–58). Notably, Patchen et al. recently conducted a meta-analysis on this clinical issue. This study included four trials, two of which specifically evaluated asthma outcomes, and found that, compared with lower or standard doses (400 IU/day), higher doses during pregnancy (2,800 IU/day; 4,400 IU/day) may have little or no effect on childhood asthma (RR 0.81, 95% CI 0.63–1.04; *I*^2^ = 29%). Both trials subsequently conducted 6-year follow-ups, and the follow-up data indicated that high-dose vitamin D intake during pregnancy may have almost no effect on childhood asthma (RR 1.21, 95% CI 0.92–1.59). However, with respect to wheezing, three studies in this meta-analysis evaluated wheezing status in 1,439 participants and found that, compared with lower or standard vitamin D doses, higher doses during pregnancy (2,800 IU/day; 4,400 IU/day; 60,000 IU every 4 weeks; 60,000 IU every 8 weeks) may reduce offspring wheezing (RR 0.79, 95% CI 0.64–0.98; *I*^2^ = 0%). These findings suggest that the potential protective effect of vitamin D on wheezing may be independent of its effect on asthma and may exhibit a more sensitive dose–response relationship (59).

Our meta-analysis of FEV1 and several secondary outcomes (FVC (%) and FEV1/FVC%) revealed substantial heterogeneity among the included studies, which may affect the robustness of the pooled estimates. Regrettably, due to the limited number of included studies, we were only able to perform subgroup analyses for FEV1, and not for secondary outcomes with higher heterogeneity, to explore the sources of heterogeneity. Subgroup analyses of FEV1 suggested that both the duration of vitamin D supplementation and participants’ baseline vitamin D status might contribute to this heterogeneity; however, varying degrees of residual heterogeneity remained within these subgroups. In addition, differences in participant characteristics and intervention contexts across studies may also influence FEV1 and other outcomes, including asthma severity and level of control, comorbidities (such as allergy-related conditions and obesity), baseline asthma treatment regimens, vitamin D administration regimen and follow-up schedules, as well as variability in the methods used for lung function measurement and reporting. Therefore, future research should aim to clarify the differential effects of vitamin D supplementation in children with asthma across diverse clinical scenarios, employing more standardized outcome measures and stratified study designs.

Our meta-analysis has several notable strengths. First, our analysis included only RCTs, a study design widely regarded as providing high-quality evidence. Second, we comprehensively evaluated the effects of vitamin D supplementation on lung function (FEV1, FVC, FEV1/FVC%, and PEF) in children with asthma and conducted multiple subgroup analyses for FEV1. Finally, in terms of data processing, to ensure methodological rigor, we analyzed outcomes using both post-intervention values and change-from-baseline values. Additionally, sensitivity analysis of the primary outcome (FEV1) indicated that our findings are robust and reliable.

Our meta-analysis also has several limitations. First, as discussed earlier, heterogeneity across studies was substantial for several outcomes, with I^2^ values exceeding 90% in some analyses. Such heterogeneity may be attributable to differences in the baseline characteristics of children with asthma, the vitamin D dosages used, and concomitant treatments other than vitamin D, thereby limiting the robustness and generalizability of the pooled estimates. Second, although the subgroup analyses suggested potential benefits of low-dose and short-duration supplementation, due to the limited sample size and the other reasons discussed above, these findings should therefore be interpreted as exploratory rather than definitive. Third, outcome measures were not fully standardized across the included studies. For example, variations in the reporting of lung function and in the timing of measurements may have further contributed to heterogeneity and limited the feasibility of direct comparisons across trials. Finally, some studies only reported that vitamin D supplementation had no significant effect on lung function in children with asthma, but did not provide analyzable data, which may have led to an overestimation of the efficacy of vitamin D in our analysis.

## 5 Conclusion

Our meta-analysis found that vitamin D supplementation in children with asthma does not improve lung function parameters (FEV1, FVC, FEV1/FVC%, PEF), IgE levels, FeNO, or cACT scores. Overall, the current evidence does not support the routine use of vitamin D supplementation to improve lung function in children with asthma.

## Data Availability

The original contributions presented in the study are included in the article/Supplementary material, further inquiries can be directed to the corresponding author.

## Footnotes

1
https://www.math.hkbu.edu.hk/~tongt/papers/median2mean.html

## Supplementary material

Supplementary Figure 1 Flowchart of the analytical process for post-intervention values and change-from-baseline values.

Supplementary Figure 2 The impact of vitamin D supplementation on FEV1 (change-from-baseline).

Supplementary Figure 3 The impact of vitamin D supplementation on FVC (change-from-baseline).

Supplementary Figure 4 The impact of vitamin D supplementation on FEV1/FVC% (change-from-baseline).

Supplementary Figure 5 The impact of vitamin D supplementation on PEF [Jat et al. (31) is change-from-baseline].

Supplementary Figure 6 The impact of vitamin D supplementation on cACT score [Jat et al. (31) is change-from-baseline].

Supplementary Figure 7 Egger’s test for primary outcome.
