# Supplementary material for: The efficacy of vitamin D supplementation in the management of childhood asthma: a systematic review and meta-analysis
Source: Front Nutr. 2026 May 19;13:1842895. doi: 10.3389/fnut.2026.1842895 (PMC13226577; doi:10.3389/fnut.2026.1842895)
Supplement: Supplementary file 1 [file Table_1.docx]

**Supplementary Table 1** Literature search process and results.

| **Databases** | **Search strategy** | **literature quantity** |
| --- | --- | --- |
| Pubmed | ((((((vitamin D) OR (25-hydroxyvitamin D)) OR (25(OH)D)) OR (cholecalciferol)) OR (vitamin D3)) OR(calcitriol)) AND (((asthma) OR (bronchial asthma)) OR (wheeze))AND ((((children) OR (child)) OR (pediatric)) OR (adolescence))AND (((random) OR (randomized)) OR (randomised))AND (((trial) OR (controlled trial)) OR (clinical trial)) | 234 |
| Web of Science | ((((((vitamin D) OR (25-hydroxyvitamin D)) OR (25(OH)D)) OR (cholecalciferol)) OR (vitamin D3)) OR(calcitriol)) AND (((asthma) OR (bronchial asthma)) OR (wheeze))AND ((((children) OR (child)) OR (pediatric)) OR (adolescence))AND (((random) OR (randomized)) OR (randomised))AND (((trial) OR (controlled trial)) OR (clinical trial)) | 262 |
| Cochrane | #1 MeSH descriptor: [Vitamin D] explode all trees  #2 MeSH descriptor: [Calcifediol] explode all trees  #3 MeSH descriptor: [Cholecalciferol] explode all trees  #4 #1 OR #2 OR #3  #5 ("vitamin D" OR "25-hydroxyvitamin D" OR "25(OH)D" OR cholecalciferol OR "vitamin D3" OR calcitriol):ti,ab,kw  #6 #4 OR #5  #7 MeSH descriptor: [Asthma] explode all trees  #8 MeSH descriptor: [Respiratory Sounds] explode all trees  #9 #7 OR #8  #10 (asthma OR "bronchial asthma" OR wheeze):ti,ab,kw  #11 #9 OR #10  #12 MeSH descriptor: [Child] explode all trees  #13 MeSH descriptor: [Adolescent] explode all trees  #14 #12 OR #13  #15 (child* OR pediatric* OR adolescent*):ti,ab,kw  #16 #14 OR #15  #17 MeSH descriptor: [Randomized Controlled Trial] explode all trees  #18 MeSH descriptor: [Clinical Trial] explode all trees  #19 #17 OR #18  #20 (random* OR randomised OR "clinical trial" OR "controlled trial"):ti,ab,kw  #21 #19 OR #20  #22 #6 AND #11 AND #16 AND #21 | 223 |
| EMbase | #1 'vitamin d'/exp OR 'calcifediol'/exp OR 'colecalciferol'/exp OR '25 hydroxyvitamin d'/exp  #2 'wheezing'/exp OR 'asthma'/exp  #3 'vitamin d':ti,ab,kw OR '25-hydroxyvitamin d':ti,ab,kw OR (25:ti,ab,kw AND oh:ti,ab,kw AND d:ti,ab,kw) OR 'cholecalciferol':ti,ab,kw OR 'vitamin d3':ti,ab,kw OR 'calcitriol':ti,ab,kw  #4 'asthma':ti,ab,kw OR 'bronchial asthma':ti,ab,kw OR 'wheeze':ti,ab,kw  #5 'children':ti,ab,kw OR 'child':ti,ab,kw OR 'pediatric':ti,ab,kw OR 'adolescence':ti,ab,kw  #6 'adolescence'/exp OR 'child'/exp OR 'pediatric'/exp  #7 'random*':ti,ab,kw OR 'randomised':ti,ab,kw OR 'clinical trial':ti,ab,kw OR 'controlled trial':ti,ab,kw  #8 'randomized controlled trial'/exp OR 'controlled study'/exp OR 'clinical trial'/exp  #9 #1 OR #3  #10 #2 OR #4  #11 #5 OR #6  #12 #7 OR #8  #13 #9 AND #10 AND #11 AND #12 | 1016 |
