# Supplementary material for: The efficacy of vitamin D supplementation in the management of childhood asthma: a systematic review and meta-analysis
Source: Front Nutr. 2026 May 19;13:1842895. doi: 10.3389/fnut.2026.1842895 (PMC13226577; doi:10.3389/fnut.2026.1842895)
Supplement: Supplementary file 2 [file Table_2.docx]

**Supplementary Table 2**. List of studies excluded at full-text screening stage, with brief reasons

| **Reasons for exlcusion** | **References** |
| --- | --- |
| Including intervention measures beyond vitamin D | 1. Xiao X, Wang R, Qin L. IMMUNE FUNCTION (SERUM IL-4 AND IL-5), NUTRITIONAL STATUS, AND CLINICAL OUTCOMES IN CHILDREN WITH BRONCHIAL ASTHMA AFTER VITAMIN D SUPPLEMENTATION. Journal of medical biochemistry. 2025;44(5):1059‐66. |
|  | 2. Zhang J, Zhang J, Shi F, Tian W, Liu X. Therapeutic effect of a combination of montelukast and vitamins A and D drops in children with bronchial asthma, and its influence on quality of life. Tropical Journal of Pharmaceutical Research. 2021;20(9):2005-11. |
|  | 3. Kang Q, Zhang X, Liu S, Huang F. Correlation between the vitamin d levels and asthma attacks in children: Evaluation of the effects of combination therapy of atomization inhalation of budesonide, albuterol and vitamin d supplementation on asthmatic patients. Experimental and Therapeutic Medicine. 2018;15(1):727-32. |
|  | 4. Miraglia Del Giudice M, Maiello N, Allegorico A, Iavarazzo L, Capasso M, Capristo C, et al. Lactobacillus reuteri DSM 17938 plus vitamin D(3) as ancillary treatment in allergic children with asthma. Ann Allergy Asthma Immunol. 2016;117(6):710-2. |
| Experimental design does not constitute a RCT | 1. Ramadan A, Sallam S, Yousef R, Elsheikh M, Ali A, Elhusseny Y, et al. Evaluation of IGF-1, TNF-α, and TGF-β Gene Expression after Oral Vitamin D Supplementation in School-Aged Children with Chronic Bronchial Asthma. Open Access Macedonian Journal of Medical Sciences. 2022;10:1358-64. |
|  | 2. Kalmarzi RN, Ahmadi S, Rahehagh R, Fathallahpour A, Khalafi B, Kashefi H, et al. The Effect of Vitamin D Supplementation on Clinical Outcomes of Asthmatic Children with Vitamin D Insufficiency. Endocr Metab Immune Disord-Drug Targets. 2020;20(1):149-55. |
|  | 3. Ramadan A, Sallam SF, Elsheikh MS, Ishak SR, Abdelsayed MGR, Salah M, et al. VDR gene expression in asthmatic children patients in relation to vitamin D status and supplementation. Gene Reports. 2019;15. |
| Study population does not meet the inclusion criteria | 1. Stefanidis C, Bush A, Newby C, Nwokoro C, Liebeschuetz S, Skene IP, et al. Vitamin D replacement in children with acute wheeze: a dose-escalation study. ERJ Open Res. 2022;8(2). |
|  | 2. Hodiatska K, Bolbot Y, Shvaratska O, Bordii T, Alifanova S. Recurrent viral-induced wheezing in young children - the protective role of vitamin D supplementation. Pediatria Polska. 2022;97(3):242-8. |
|  | 3. de Groot JC, van Roon ENH, Storm H, Veeger N, Zwinderman AH, Hiemstra PS, et al. Vitamin D reduces eosinophilic airway inflammation in nonatopic asthma. J Allergy Clin Immunol. 2015;135(3):670-U133. |
| The experimental design is a RCT but does not meet the inclusion criteria | 1. Alansari K, Davidson BL, Holick MF. A randomized comparison of intramuscular high-dose versus oral maintenance vitamin D to prevent severe exacerbations in deficient/insufficient asthmatic children. J Allergy Clin Immunol Glob. 2025;4(3):100497. |
|  | 2. Alansari K, Davidson BL, Yousef KI, Mohamed ANH, Alattar I. Rapid vs Maintenance Vitamin D Supplementation in Deficient Children With Asthma to Prevent Exacerbations. Chest. 2017;152(3):527-36. |
| No outcome indicators meet the inclusion criteria | 1. Swangtrakul N, Manuyakorn W, Mahachoklertwattana P, Kiewngam P, Sasisakulporn C, Jotikasthira W, et al. Effect of vitamin D on lung function assessed by forced oscillation technique in asthmatic children with vitamin D deficiency: A randomized double-blind placebo-controlled trial. Asian Pac J Allergy Immunol. 2022;40(1):22-30. |
|  | 2. Jensen ME, Mailhot G, Alos N, White JH, Rousseau E, Khamessan A, et al. A vitamin D intervention in preschoolers with viral-induced asthma: a pilot randomised contorlled trial (DIVA). Am J Respir Crit Care Med. 2015;191(Meeting Abstracts):A3360. |
|  | 3. Yadav M, Mittal K. Effect of vitamin D supplementation on moderate to severe bronchial asthma. Indian J Pediatr. 2014;81(7):650-4. |
| Different reports on the same study | 1. Kamran M, Naqvi N, Khan M, Kumar S, Soomro AK, Khalil S. EFFECT OF VITAMIN D3 SUPPLEMENTATION ON SEVERE ASTHMA EXACERBATIONS IN CHILDREN WITH ASTHMA AND LOW VITAMIN D LEVELS: THE VDKA RANDOMIZED CLINICAL TRIAL. NeuroQuantology. 2023;21(6):1448‐57. |
|  | 2. Forno E, Bacharier LB, Phipatanakul W, Guilbert TW, Cabana M, Ross KR, et al. Vitamin d supplementation and severe disease exacerbations in children with asthma and low vitamin d levels: a randomized controlled trial. Am J Respir Crit Care Med. 2020;201(1). |
|  | 3. Kerley CP, Hutchinson K, Greally P, Coghlan D, Elnazir B. A randomized, double-blind, placebo-controlled of vitamin D3 for Irish children with asthma. Proceedings of the Nutrition Society. 2015;74(OCE4). |
|  | 4. Kerley CP, Hutchinson K, Greally P, Coghlan D, Elnazir B. The effects of vitamin d supplementation on pulmonary function, disease severity and markers of inflammation in childhood asthmatics: a randomized, double-blind, placebo-controlled trial. Irish journal of medical science. 2014;183(11):S522. |
