# Supplementary figures and images for: The efficacy of vitamin D supplementation in the management of childhood asthma: a systematic review and meta-analysis
Source: Front Nutr. 2026 May 19;13:1842895. doi: 10.3389/fnut.2026.1842895 (PMC13226577; doi:10.3389/fnut.2026.1842895)

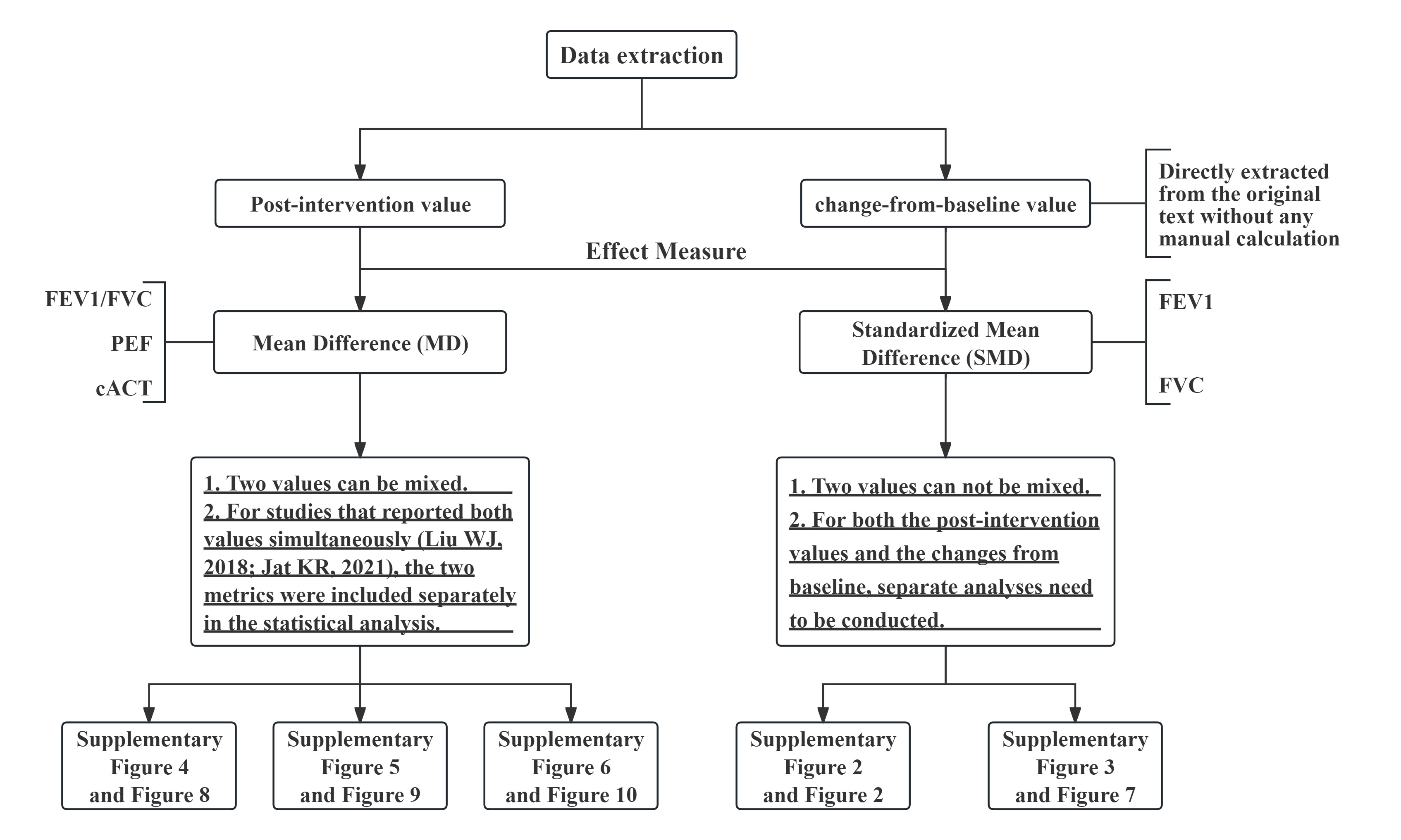

Supplement: Supplementary Figure 1 — Flowchart of the analytical process for post-intervention values and change-from-baseline values. [file Image_1.jpeg]

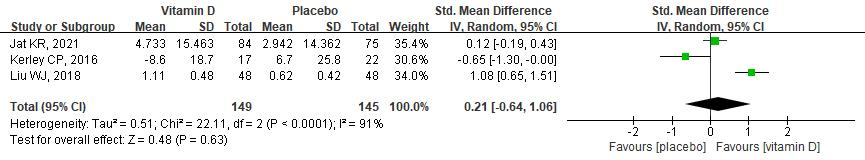

Supplement: Supplementary Figure 2 — The impact of vitamin D supplementation on FEV1 (change-from-baseline). [file Image_2.jpeg]

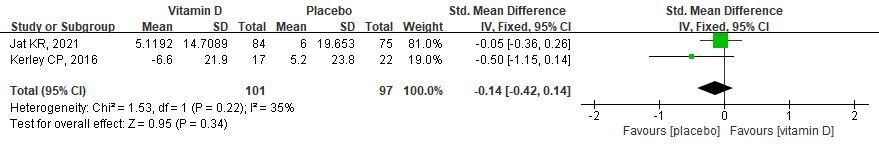

Supplement: Supplementary Figure 3 — The impact of vitamin D supplementation on FVC (change-from-baseline). [file Image_3.jpeg]

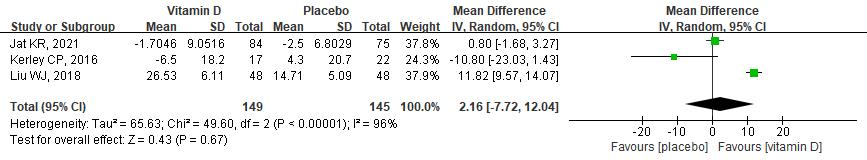

Supplement: Supplementary Figure 4 — The impact of vitamin D supplementation on FEV1/FVC% (change-from-baseline). [file Image_4.jpeg]

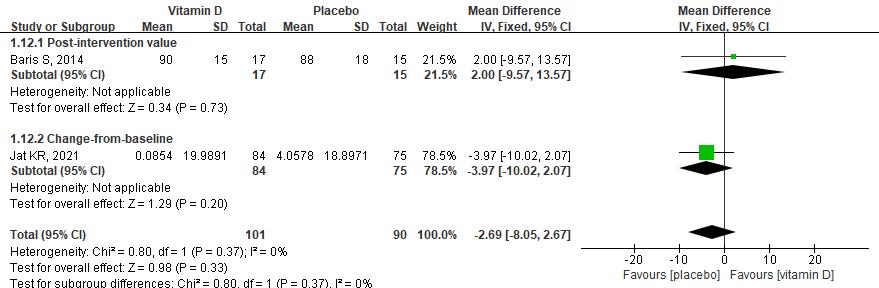

Supplement: Supplementary Figure 5 — The impact of vitamin D supplementation on PEF [Jat et al. (31) is change-from-baseline]. [file Image_5.jpeg]

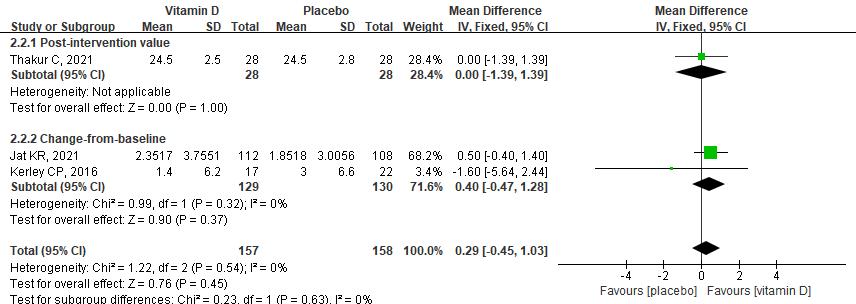

Supplement: Supplementary Figure 6 — The impact of vitamin D supplementation on cACT score [Jat et al. (31) is change-from-baseline]. [file Image_6.jpeg]

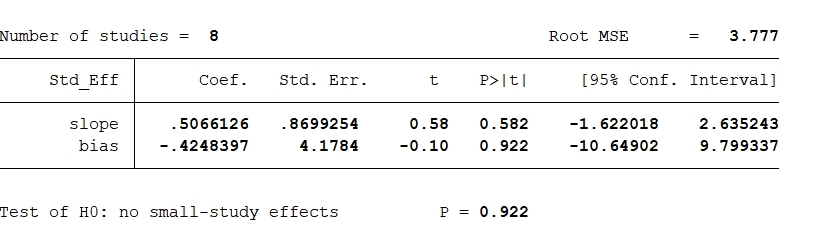

Supplement: Supplementary Figure 7 — Egger’s test for primary outcome. [file Image_7.jpeg]
